# Preliminary comp arison of helical tomotherapy and mixed beams of unmodulated electrons and intensity modulated radiation therapy for treating superficial cancers of the parotid gland and nasal cavity

**DOI:** 10.1186/1748-717X-6-178

**Published:** 2011-12-28

**Authors:** Olivier Blasi, Jonas D Fontenot, Robert S Fields, John P Gibbons, Kenneth R Hogstrom

**Affiliations:** 1 Department of Physics and Astronomy, Louisiana State University and Agricultural and Mechanical College, Baton Rouge, LA, USA; 2 Department of Physics, Mary Bird Perkins Cancer Center, Baton Rouge, LA, USA

**Keywords:** electron beam therapy, intensity modulated radiation therapy, helical tomotherapy, mixed beam therapy, head and neck cancer

## Abstract

**Background and Purpose:**

To investigate combining *unmodulated *electron beams with intensity-modulated radiation therapy to improve dose distributions for superficial head and neck cancers, and to compare mixed beam plans with helical tomotherapy.

**Materials and methods:**

Mixed beam and helical tomotherapy dose plans were developed for two patients with parotid gland tumors and two patients with nasal cavity tumors. Mixed beam plans consisted of various weightings of a enface electron beam and IMRT, which was optimized after calculation of the electron dose to compensate for heterogeneity in the electron dose distribution within the target volume.

**Results:**

Helical tomotherapy plans showed dose conformity and homogeneity in the target volume that was equal to or better than the mixed beam plans. Electron-only plans tended to show the lowest doses to normal tissues, but with markedly worse dose conformity and homogeneity than in the other plans. However, adding a 20% IMRT dose fraction (*i.e*., IMRT:electron weighting = 1:4) to the electron plan restored target conformity and homogeneity to values comparable to helical tomotherapy plans, while maintaining lower normal tissue dose.

**Conclusions:**

Mixed beam treatments offer some dosimetric advantages over IMRT or helical tomotherapy for target depths that do not exceed the useful range of the electron beam. Adding a small IMRT component (*e.g*., IMRT:electron weighting = 1:4) to electron beam plans markedly improved target dose homogeneity and conformity for the cases examined in this study.

## I. Introduction

Clinical radiation therapy techniques for superficial (*i.e*., located within approximately 5 cm of the patient surface) head and neck cancers have evolved dramatically over the past several decades. Historically, superficial head and neck targets were treated with wedged pairs of photons, orthovoltage x-rays, or electron beams often mixed with photons [1]. Introduction of the modern photon multileaf collimator (MLC) enabled intensity modulated delivery techniques that improved dose conformity and homogeneity compared to electron and conventional mixed techniques [2], resulting in better tumor control and lower normal tissue complication probabilities. A more recent development is helical tomotherapy, where intensity modulation is achieved by translating the patient through a rotating gantry on which a linear accelerator head and binary MLC are mounted [3].

Previous works that have examined helical tomotherapy for superficial cancers generally conclude that it is capable of delivering dose distributions that are equivalent to or better than distributions delivered with fixed-beam IMRT [4,5] These results, augmented with institutional experience, have resulted in helical tomotherapy often being the treatment of choice for head and neck cancer in our clinic.

A number of groups have reported dosimetric results from intensity- and energy- modulated electron beams using either a commercial photon [6-9] or custom electron [10-12] MLC. For example, Salguero *et al. *[8] reported that modulated electron radiation therapy plans created with a photon MLC showed comparable target volume dose homogeneity and significantly lower normal tissue dose compared with fixed-beam IMRT plans in four patients with superficial tumors head and neck tumors. Ma *et al. *[13] concluded that modulated electron therapy could achieve superior dose conformity for superficial breast cancer compared with IMRT. However, those studies required either custom hardware (in the case of electron MLC) and/or custom treatment planning systems (typically configured with Monte Carlo simulation capabilities) that are not widely available. Other studies have reported on combining *unmodulated *electron beams with IMRT to improve dose distributions for head and neck cancers. Mu *et al. *[14] compared various mixtures of electrons and IMRT for five deep-seated head and neck targets. They reported that the mixed beam plans achieved adequate target and normal tissue dose with lower integral dose, and concluded that mixed beam plans could more readily meet planning goals if computerized optimization tools were available. Their study was limited in that (1) some or all of the selected target structures typically exceeded the useful range of electron beams and (2) inverse planning of the IMRT fields did not include dose contributions from the electron beams. More recently, Chan *et al. *reported that combining electrons and IMRT improved target dose homogeneity and decreased normal tissue dose for patients with extensive scalp lesions [15] and mesothelioma [16] compared with IMRT alone.

The motivation for this study was (1) to assess the utility of combining unmodulated electron beams with IMRT for patients with disease that could best exploit the advantages of electron beams, and (2) to determine the optimal beam weighting when IMRT fields are allowed to optimize with the electron beam dose taken into account. Our objective was to investigate mixed beam plans for treating superficial cancers of the parotid gland and nasal cavity, and to compare these plans with those developed with our current clinical standard of care for head and neck cancer, helical tomotherapy.

## II. Methods and materials

### II.A. Patients

Four patients previously treated at our institution for superficial head and neck cancers were selected and placed into a Health Insurance Portability and Accountability-compliant database. The criteria for patient selection was (1) that the primary planning target volume (PTV) did not extend beyond the therapeutic range of a 20 MeV electron beam (approximately 5.5 cm in tissue) (2) the availability of three-dimensional computed tomography data suitable for treatment planning and (3) the patient had been previously treated with IMRT or TomoTherapy. Final patient selection was determined by the physician on the basis of clinical interest in site that stood to benefit most from mixed beam treatments. Disease pathology, staging (where available), location, patient gender, and patient age for the patients selected for this work are shown in Table 1. Helical tomotherapy and mixed beam treatment plans were developed for these patients and compared.

**Table 1:** Relevant disease data for patients selected in the study

Patient	Diagnosis	Staging	Disease Location	Age	PTV volume
1	Intermediate grade mucoepidermoid carcinoma	T1N0M0	Right parotid	53	355.4 cm^3^
2	Squamous cell carcinoma	Unstaged	Left parotid	80	197.7 cm^3^
3	Moderately differentiated Squamous cell carcinoma	T1-2N0M0	Left nasal septum	73	57.6 cm^3^
4	Squamous cell carcinoma	cT1N0M0	Left nasal septum	90	28.7 cm^3^

### II.B. Treatment planning

#### PTVs and OARs/prescriptions

Organ-at-risk (OAR) volumes were contoured for each each patient. Most OARs were generated by either the physician or the dosimetrist (evaluated and modified by the physician if necessary) in the original plan. The OARs previously contoured for the parotid patients were the contralateral parotid, eyes, lenses, optic nerve, and spinal cord. The OARs previously contoured for the nasal cavity patients were the spinal cord, eyes, lenses, and optic nerves.

For the parotid gland tumors, in order to provide a build-up of dose to the PTV near the skin surface or to surgical scars in these post-operative patients, a tissue-equivalent bolus was contoured. The bolus also served to compensate for missing tissue, around the ear. The bolus for the parotid patients was either contoured as a 0.5 cm or 1.0 cm thick (depending on the depth of PTV, energy of electron beam, and OAR distal to the PTV) expansion to the skin. Bolus was also used for the nasal cavity tumors to minimize the impact of surface irregularities on the electron dose. For these patients, the bolus was fabricated during simulation such that an enface electron beam would impinge upon a flat surface.

Once all the contours and dose limiting structures were defined, pre-optimization plan parameters values were set. The prescription and planning goals in the helical tomotherapy and mixed beams plans were kept as close as the differences in the systems would allow. Dose prescriptions for the parotid gland tumors were 59.4 Gy and 65.0 Gy for patients 1 and 2, respectively; nasal cavity tumors were 70.0 Gy and 65.0 Gy for patients 3 and 4, respectively.

#### Mixed Beam Planning

Mixed beam plans were constructed using the Philips Pinnacle^3 ^(8.1×, Philips Medical Systems, Fitchburg, WI) treatment planning system with corrections for patient heterogeneities. Mixed beam plans were designed as a combination of a five (nasal cavity patients) or seven (parotid gland patients) field 6-MV IMRT plan optimized on top of a single enface 16 MeV or 20 MeV electron beam. For all patients, the enface electron beam was oriented normal to the patient plane and blocked to a 1 cm margin around the PTV. A 1.0 cm thick lead skin collimation was contoured over the eyes of the nasal cavity patients to minimize electron scatter. For the nasal cavity patients, the IMRT fields were evenly spaced every 70 degrees. For the parotid gland patients, the IMRT fields were arranged around the ipsilateral side of body approximately every 30 degrees.

Because the ideal weighting (*i.e*., the ratio of photon dose to electron dose) was not known, seven different ratios of IMRT to electron dose were investigated. Dose weighting was specified as the ratio of absorbed dose from the IMRT component to that from the electron component at the point of maximum dose in the electron beam. The following dose weights were investigated: 1:0 (IMRT only), 2:1, 1:1, 1:2, 1:3, 1:4, and 0:1 (electron only). For those plans utilizing a combination of IMRT and electrons, the electron dose component was calculated *prior *to IMRT optimization to allow for the IMRT dose component to compensate for heterogeneity in the electron dose distribution within the PTV.

#### Helical Tomotherapy Planning

Helical tomotherapy plans were constructed using TomoTherapy Hi-Art treatment planning system (version 3.1.2). Plan optimization was performed in beamlet mode, where a beamlet is the fluence through a single-leaf opening at 1 projection. There are 64 binary leaves (0.625 cm wide at isocenter) for each projectuib, and 51 projection for each gantry rotation. During optimization, the weights of all beamlets that intersect PTVs are adjusted to achieve user-defined volumetric dose goals. Plans were generated using parameters typical of clinical delivery at the time of the study, including a 2.5-cm nominal jaw opening and a pitch (fraction of the jaw opening advanced by the treatment couch per revolution) of 0.287. The planning modulation factor (the ratio of the highest beamlet intensity to the average intensity of all nonzero beamlets) was set to a maximum of 3, which is the standard value used in our clinic. TomoTherapy dose distributions were transferred to the Pinnacle^3 ^system for comparison to those of the mixed-beam technique

### II.C. Plan evaluation

Helical tomotherapy and mixed beam treatments plans were evaluated on the basis of dosimetric endpoints: target volume coverage, conformity index (CI), dose homogeneity index (DHI) for targets; maximum dose, mean dose, and select dose-volume metrics for OARs. CI was computed for the PTV using the method of Paddick [17], given by

(1) $$\text{CI = }\frac{{\text{TV}}_{\text{PIV}}^{\text{2}}}{\text{TV}\times \text{PIV}}$$

where TV_PIV _represents the volume of the PTV within the prescription isodose line, TV denotes the volume of the PTV, and PIV denotes the volume encompassed by the prescription isodose line. Values of CI are dimensionless, have a value of 1 for an ideal treatment, and decreases as the prescription isodose line encompasses larger volumes outside the PTV. The dose homogeneity index (DHI) was also computed for the PTV such that

(2) $$\text{DHI = }\frac{{\text{D}}_{\text{max}}-{\text{D}}_{\text{min}}}{{\text{D}}_{\text{Rx}}}$$

where D_max _represents the dose to 1% of the PTV, D_min _denotes the dose to 99% of the PTV, and D_Rx _denotes the prescribed dose. Values of DHI are dimensionless, have a value of 0 for an ideal treatment, and increase as dose to the PTV becomes less uniform.

Helical tomotherapy and mixed beam plans were also compared using radiobiological metrics: normal tissue complication probability (NTCP) and tumor control probability (TCP). NTCP values were computed using the Lyman-Kutcher-Burman probit model [18,19]. TCP values were computed for the PTV using the standard Poisson dose-response model [20] assuming homogeneous tumor cell distribution. Additional details regarding radiobiological parameters used for calculation of NTCP and TCP can be found elsewhere [21,22].

## III. Results

**Figure 1:**
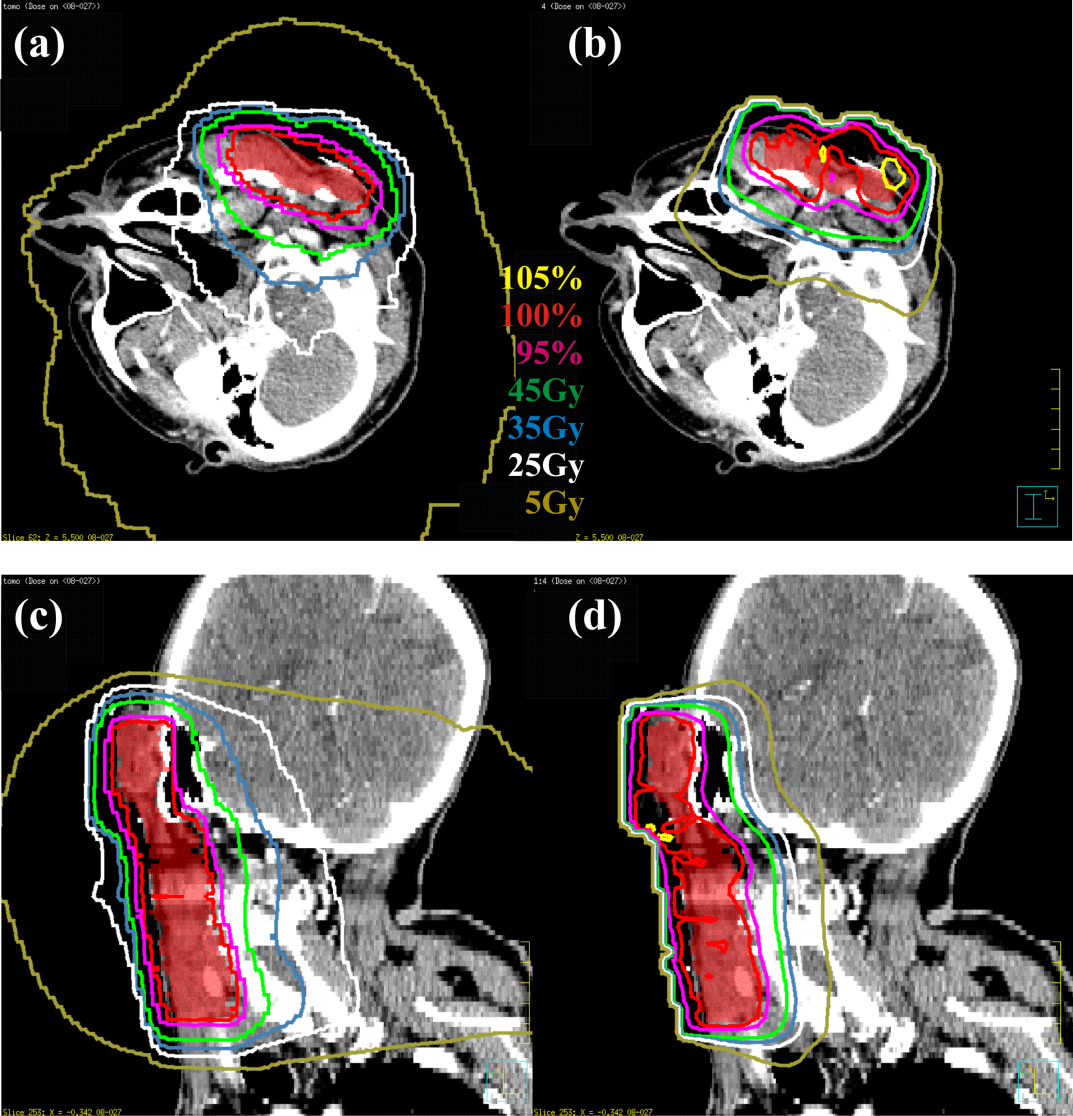
**Isodose distributions for a TomoTherapy plan (left) and mixed beam plan (IMRT:electron weighting = 1:4, right) of a patient with cancer of the parotid gland**. The PTV is shown in red colorwash.

**Figure 2:**
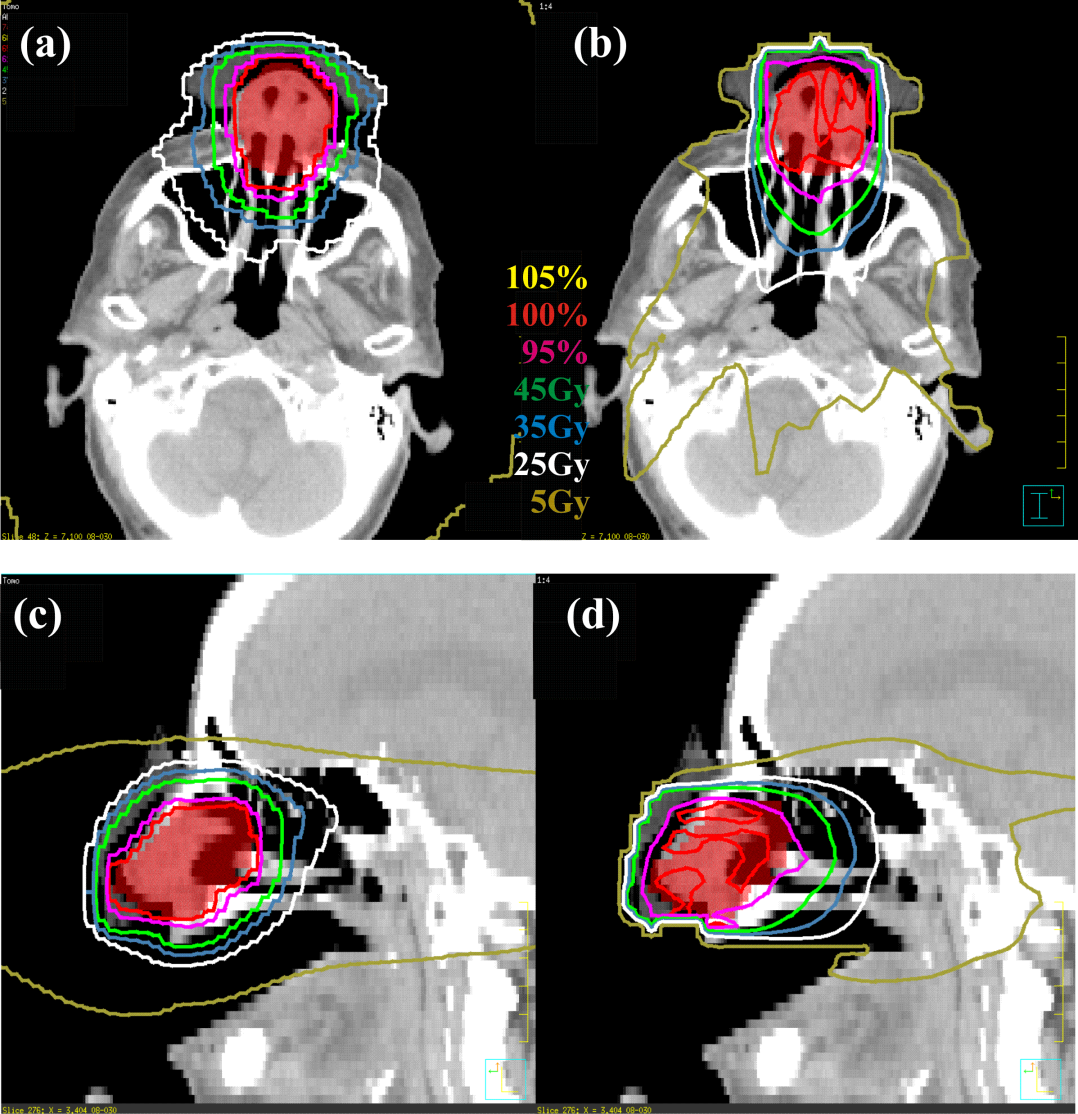
**Isodose distributions for a TomoTherapy plan (left) and mixed beam plan (IMRT:electron weighting = 1:4, right) of a patient with cancer of the parotid gland**. The PTV is shown in red colorwash.

Figures 1 and 2 show isodose plots in the axial and sagittal planes for patients 2 (parotid gland) and 4 (nasal cavity) for helical tomotherapy and a selected mixed beam plan. The PTV volume is visible as a red colorwash contour, along with isodose lines indicating the desired prescription dose levels. While both plans demonstrated good coverage of the PTV, the helical tomotherapy plans showed greater dose homogeneity within the PTV and a better conformance of the 100% isodose line to the PTV. The mixed beam plan showed marked reductions in volumes of tissue encompassed by lower isodose lines (*e.g*., 35, 25, and 5 Gy). Similar results were seen in the other patients.

**Figure 3:**
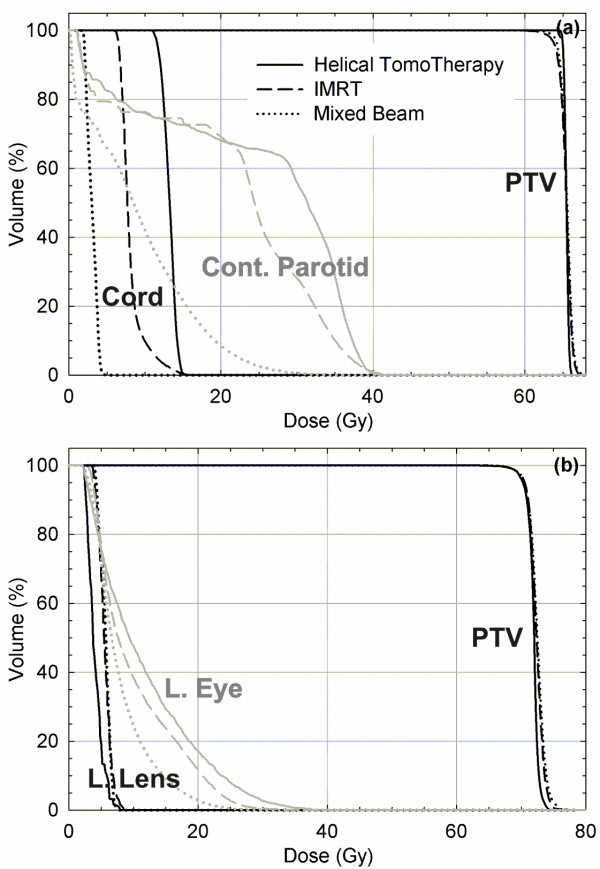
**DVHs for (a) patient 2 (parotid gland) and (b) patient 3 (nasal cavity) from helical tomotherapy, IMRT, and mixed beam (1:4 IMRT-electron weighting)**. *Abbreviations: *PTV = planning target volume; Cont. = contralateral; L. = left.

Figure 3 shows DVHs for patients 2 (parotid gland) and 3 (nasal cavity) for three plans: helical tomotherapy (solid lines), IMRT (dashed lines), and mixed beam (dotted line, 1:4 IMRT-electron weighting). For the both patients, the helical tomotherapy plan show better dose uniformity in the target volume, with the IMRT and mixed beam plans showing similar PTV coverage and uniformity. For the parotid gland patient, the mixed beam plan showed substantial reduction to the spinal cord and contralateral parotid gland over all dose intervals. For the nasal cavity patient, the mixed beam plan showed better sparing of the left eye, while all three plans showed similar dose to the left lens. A detailed description of target homogeneity, critical structure involvement, biological results for the plans examined in this work is now presented.

### III.A. Parotid Gland Patients

Conformity indices (CI), dose homogeneity indices (DHI), and tumor control probabilities (TCP) for each parotid gland patient for all plans examined in this work are shown in Table 2. In both patients, the DHI was best for the helical tomotherapy plan and worst for the electron-only plan. DHI values were similar amongst IMRT and mixed beam plans, being 0.03-0.04 larger than those for helical tomotherapy plans. Similarly, CI was best for the helical tomotherapy plan, worst for the electron-only plan, and similar among the IMRT and mixed beam plans. The CI value in the helical tomotherapy plan was comparable to the mixed beam plans in patient 1, but were 0.07-0.25 greater in patient 2. Of note was that the introduction of a small IMRT component into the electron plan (*e.g*., the 1:4 mixed beam plan) restored the CI and DHI to values comparable to the IMRT plan, *i.e*., adding additional IMRT was of no benefit. Despite the differences in CI and DHI, the TCP values were greater than 98.4% for all plans and less than 1% variation amongst the different plans, with the exception of the electron-only plan for Patient 1.

**Table 2:** Conformity index (CI), dose homogeneity index (DHI), tumor control probability (TCP), normal tissue complication probably (NTCP) and normal tissue dose metrics for the parotid gland patients.

Patient	Plan (IMRT:e)	PTV			Normal Tissues					
		CI	DHI	TCP (%)	*Cont. Parotid*		*Spinal Cord*		*Cont. Eye*	*Ips. Eye*
					D_mean_ (Gy)	NTCP (%)	D_max_ (Gy)	NTCP (%)	D_max_ (Gy)	D_max_ (Gy)
1	HT	0.85	0.06	98.5	6.0	6.8	43.2	0.3	6.9	9.1
	1:0	0.84	0.10	98.6	7.3	7.9	45.3	0.5	2.9	4.4
	2:1	0.85	0.09	98.7	6.7	7.3	46.6	0.6	3.2	4.0
	1:1	0.80	0.10	98.6	6.9	7.5	48.1	0.5	3.6	4.1
	1:2	0.83	0.09	98.7	5.0	6.0	43.8	0.4	3.5	3.7
	1:3	0.82	0.09	98.7	4.9	6.0	44.2	0.4	3.5	3.7
	1:4	0.81	0.10	98.7	4.9	5.9	43.9	0.3	3.6	3.8
	0:1	0.66	0.27	95.6	3.4	4.9	45	0.4	3.6	3.9

2	HT	0.77	0.02	100	13.2	14.4	41.2	0.4	12	11.9
	1:0	0.65	0.06	100	8.2	8.6	43.3	0.3	7.6	11.1
	2:1	0.65	0.06	100	6.0	6.8	39	0.1	6.2	13.5
	1:1	0.70	0.05	100	5.1	6.1	37.2	< 0.1	4.7	13.7
	1:2	0.52	0.06	100	4.0	5.3	36.1	< 0.1	3.8	14.1
	1:3	0.60	0.06	100	3.5	5.0	35.8	< 0.1	3.0	14.0
	1:4	0.59	0.05	100	3.1	4.8	35.6	< 0.1	3.7	16.0
	0:1	0.18	0.18	99.7	1.1	3.7	30.7	< 0.1	1.6	14.6

Beam weightings indicate the ratio of IMRT-to-electron dose of the treatment plans. *Abbreviations: *PTV = planning target volume; Cont. = contralateral; Ips. = ipsilateral; R. = right; L. = left.

Selected dose metrics and normal tissue complication probabilities (NTCP) for critical structures are shown in Table 2 for all plans. In both patients, mean dose to the contralateral parotid and the corresponding NTCP was lowest in the electron-only plan. Mean dose and NTCP for the contralateral parotid were largest in the IMRT plan (7.3 Gy and 7.9%, respectively) in Patient 1, and in the helical tomotherapy plan (13.2 Gy and 14.4%, respectively) in Patient 2. In general, mean dose and NTCP for the contralateral parotid decreased as electron weighting increased. Maximum dose to the spinal cord was comparable amongst all plans, and the corresponding NTCP did not exceed 0.6% in any plan. Maximum dose to the eyes was greatest in the helical tomotherapy plans (range: 6.9 - 12 Gy), with the exception of the ipsilateral eye in Patient 2.

### III.B. Nasal Cavity Patients

CI, DHI, TCP for each nasal cavity patient are shown in Table 3 for all plans. As with the parotid gland patients, the DHI was best for the helical tomotherapy plan and worst for the electron-only plan. DHI values were similar amongst IMRT and mixed beam plans, and were 0.02-0.06 larger than in the helical tomotherapy plans. CI was comparable among all plans in Patient 3, and best in the helical tomotherapy plan in Patient 4. As was noted previously, the introduction of a small IMRT component into the electron plan restored the CI and DHI to values comparable to the IMRT plan. TCP values were greater than 99.9% or higher for all plans.

**Table 3:** Conformity index (CI), dose homogeneity index (DHI), tumor control probability (TCP), normal tissue complication probably (NTCP) and normal tissue dose metrics for the nasal cavity patients.

Patient	Plan (IMRT:e)	PTV			Normal Tissues					
		CI	DHI	TCP (%)	*L. Lens*		*R. Lens*		*L. Eye*	*R. Eye*
					D_mean_ (Gy)	NTCP (%)	D_max_ (Gy)	NTCP (%)	D_max_ (Gy)	D_max_ (Gy)
3	HT	0.80	0.07	100	8.0	0.3	14.9	2.8	40.5	37.6
	1:0	0.76	0.09	100	8.9	0.6	9.9	0.6	36.0	39.2
	2:1	0.84	0.09	100	11.0	2.2	9.6	0.8	32.1	33.2
	1:1	0.85	0.10	100	10.6	2.6	9.8	0.9	32.3	33.8
	1:2	0.85	0.11	100	8.8	0.6	8.0	0.4	27.5	32.3
	1:3	0.83	0.09	100	6.6	0.3	8.5	0.4	28.4	33.5
	1:4	0.82	0.10	100	7.6	0.6	9.2	0.5	30.2	33.0
	0:1	0.75	0.29	99.9	4.3	0.1	7.8	0.2	22.7	30.0

4	HT	0.80	0.04	100	2.9	0.1	3.5	0.1	6.4	8.6
	1:0	0.75	0.14	100	3.5	0.1	4.0	0.1	22.0	9.1
	2:1	0.60	0.07	100	2.8	0.1	4.0	0.1	12.3	9.1
	1:1	0.72	0.08	100	3.3	0.1	4.4	0.1	12.7	8.7
	1:2	0.68	0.08	100	3.3	0.1	3.9	0.1	12.0	10.1
	1:3	0.79	0.10	100	3.6	0.1	3.9	0.1	10.1	7.8
	1:4	0.73	0.09	100	2.7	0.1	3.7	0.1	10.6	9.7
	0:1	0.57	0.19	100	1.8	< 0.1	2.3	< 0.1	17.1	8.4

Beam weightings indicate the ratio of IMRT-to-electron dose of the treatment plans. *Abbreviations: *PTV = planning target volume; Cont. = contralateral; Ips. = ipsilateral; R. = right; L. = left.

Selected dose metrics and NTCP values for critical structures are shown in Table 3 for all plans. In both patients, maximum dose to the lenses of the eyes was lowest in the electron-only plans (1.8 - 7.8 Gy). Maximum dose to the eyes was also lowest in the electron-only plans, with the exception of the left eye and Patient 4. The IMRT and mixed beam plans tended to show similar normal tissue doses. Helical tomotherapy plans showed the highest lens and eye doses in Patient 3, and doses lower than or comparable to the mixed beam plans in Patient 4. NTCP values did not exceed 2.8% in any plan.

## IV. Discussion

In this study we compared helical tomotherapy and IMRT-electron mixed beam plans of various weightings (including IMRT-only and electron-only) for superficial head and neck cancers of the parotid gland and the nasal cavity. Helical tomotherapy plans showed dose conformity and homogeneity in the PTV that was equal to or better than IMRT and mixed beam plans. Electron-only plans tended to show the lowest doses to normal tissues, particularly in the parotid gland patients where critical structures were located further from the PTV, but with markedly worse dose conformity and homogeneity in the target volume than in the other plans. However, adding a 20% IMRT dose fraction (*e.g*., IMRT:electron weighting = 1:4) to the electron plan restored target conformity and homogeneity to values equal to or better than the IMRT-only plans and comparable to helical tomotherapy plans, while only slightly increasing normal tissue dose. These results suggest that (1) IMRT-electron mixing offers some dosimetric advantages over IMRT or helical tomotherapy for target depths do not exceed the useful range of the electron beam and (2) only a small (20% in this study) component of IMRT is needed to restore dose homogeneity to the target volume in an electron plan.

The results of this work are consistent with the studies from Mu *et al. *[14] and Chan *et al. *[15,16] that investigated the use of combining unmodulated electron beams and IMRT for head and neck targets. Both studies also reported that the mixed beam approach could achieve dose homogeneity and normal tissue sparing comparable to IMRT plans. However, our study further indicated that mixed beams of electrons and IMRT can produce dosimetric results comparable to helical tomotherapy, which has generally been shown to provide superior dosimetric results for head and neck cancers compared with IMRT alone. We believe the improvement in dosimetric results can be attributed to our approach of allowing the IMRT optimization to account for the electron dose component during treatment planning.

**Figure 4:**
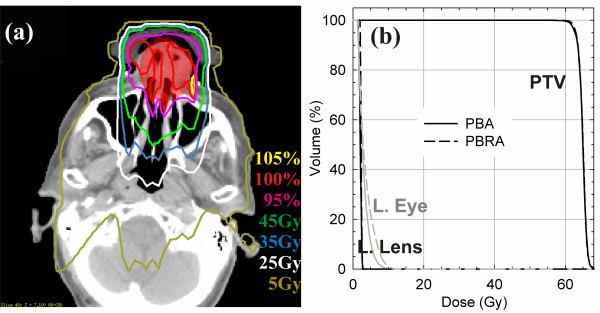
**Transverse view of the dose distribution taken through the PTV (shown in red colorwash) (a) and DVHs (b) for the mixed beam (1:4 IMRT-electron weighting) of patient 3 where the electron dose has been calculated with the pencil beam redefinition algorithm (PBRA)**. The mixed beam plan calculated with the PBRA showed negligible differences compared with the original plan, which was calculated using the electron PBA. *Abbreviations: *PTV = planning target volume; L. = left.

The results of this work are further expected to be independent of the electron dose calculation algorithm employed. In this work, the electron beam dose was calculated using the pencil beam algorithm (PBA), which has been shown to produce significant errors in regions of tissue heterogeneity [23,24]. While use of a different electron dose calculation algorithm may change the shape of the electron dose distribution, these dosimetric changes can be accounted for with the IMRT component so long as it is optimized on top of the electron dose. To verify our assertion, the mixed beam plan (1:4 IMRT-electron weighting) for patient 3 was reoptimized following calculation of the electron beam dose using the pencil beam redefinition algorithm (PBRA), which has been shown to significantly improve electron dose calculation accuracy in heterogeneous phantoms [25,26]. As shown in Figure 4, differences between plans optimized and calculated with the PBA and PBRA are negligible.

Mixed beam therapy has some practical concerns when compared with IMRT or helical tomotherapy that need consideration. First, clinical implementation of mixed beam therapy using conventional, existing techniques requires additional setup time. Delivery of the electron component requires use of an electron applicator and is typically treated using an SSD technique, whereas delivery of the IMRT component requires removal of the electron applicator and is typically treated using an SAD technique. As such, radiation technologists are required to enter the treatment room to adjust the accelerator hardware and reset the patient couch prior to delivery of the next modality. Future work should investigate the use of conventional photon MLCs to shape and deliver electron fields, which would eliminate the need for the radiation technologist to enter the room between modality deliveries. Furthermore, because the IMRT component of the mixed beam plan was used to compensate for the heterogeneity of the electron dose distribution, the IMRT component was also heterogeneous. Dose gradients within a given modality could introduce hot or cold spots in composite dose distribution in the presence of any misalignment between modality deliveries. Finally, use of current commercial electron pencil beam algorithms for mixed beam planning must be validated and, as such, our group is currently investigating dosimetric verification of mixed beam plans [27].

Recently, electron conformal therapy using a variable wax bolus has become widely available [28,29], which can produce dose distributions superior to open electron fields. Future work should attempt to investigate the use of a variable thickness wax bolus, which should even further reduce normal tissue dose with comparable dose homogeneity in the mixed beam plans.

## V. Conclusion

Our results suggest that mixed beam treatments offer dosimetric advantages over IMRT or helical tomotherapy for target depths that do not exceed the useful range of the electron beam. Adding a small IMRT component (*e.g*., IMRT:electron weighting = 1:4) to electron beam plans markedly improved target dose homogeneity and conformity for the cases examined in this study.

## Competing interests

This work was supported in part by a research agreement with TomoTherapy, Inc. However, TomoTherapy, Inc., did not participate in the study design; in the collection, analysis, and interpretation of data; in the writing of the manuscript; or in the decision to submit the manuscript for publication.

## Authors' contributions

OB constructed treatment plans and acquired the data. RF identified patients and evaluated treatment plans. OB and JF analyzed the data. All authors participated in the design of the study, and approved the final manuscript.
